# Docosahexaenoyl ethanolamide mitigates IgE-mediated allergic reactions by inhibiting mast cell degranulation and regulating allergy-related immune cells

**DOI:** 10.1038/s41598-019-52317-z

**Published:** 2019-11-07

**Authors:** Kosuke Nishi, Yoshiki Kanayama, In-Hae Kim, Akihiro Nakata, Hisashi Nishiwaki, Takuya Sugahara

**Affiliations:** 1 Department of Bioscience, Graduate School of Agriculture, Ehime University, Matsuyama, Ehime 790-8566 Japan; 2 Food and Health Sciences Research Center, Ehime University, Matsuyama, Ehime 790-8566 Japan; 3 Research Unit for Skeletal Health and Diseases, Ehime University, Toon, Ehime 791-0295 Japan; 4 Department of Pathophysiology, Graduate School of Medicine, Ehime University, Toon, Ehime Japan

**Keywords:** Nutrition, Quality of life

## Abstract

Docosahexaenoic acid (DHA) is a long-chain polyunsaturated fatty acid mainly found in fish oil. Although several studies have suggested that it can alleviate allergy symptoms, its mechanism of action remains to be elucidated. In the present study, we found that docosahexaenoyl ethanolamide (DHEA), a metabolite of DHA produced in the human body, exerts the anti-allergic activity *in vitro* and *in vivo*. DHEA suppressed degranulation of rat basophilic leukemia RBL-2H3 cells and bone marrow-derived mast cells in a dose-dependent manner without cytotoxicity. This occurred due to a decrease in Ca^2+^ influx, which is critical for mast cell degranulation. DHEA also suppressed IgE-mediated passive cutaneous anaphylaxis reaction in mice. In addition, DHEA was demonstrated to lessen an allergic symptom in a mouse model of pollinosis and to alter the production of IgE and cytokines secreted by splenocytes collected from the pollinosis mice. Taken together, this study indicates that DHEA is a promising anti-allergic agent as it inhibits mast cell degranulation and modulates other immune cells.

## Introduction

The prevalence of allergic diseases has been increasing worldwide over the last few decades. For example, the percentage of the population with seasonal allergic rhinitis, one of the most common allergic diseases in the population of Japan, has dramatically increased from 19.6% surveyed in 1998 to 29.8% in 2008^1^. Similarly, in Europe, at least 25% of school-age children have an allergic disease^2^. Allergic diseases arise as a consequence of IgE-mediated hypersensitivity to a specific allergen^3^ and are often associated with an impaired quality of life. Therapeutic agents commonly used to treat IgE-mediated allergic diseases include antihistamines, mast cell stabilizers, and other drugs. Although antihistamines are the most commonly prescribed drug for ameliorating allergic symptoms, they sometimes cause adverse effects such as drowsiness and headaches^4^. Allergen immunotherapy is also a potential treatment for allergies, although there is a risk of serious adverse effects and the exact mechanisms underlying allergen immunotherapy are still not completely elucidated. Recently, food ingredients with anti-allergy activity are attracting attention because they can be safely used long term with no or little adverse effects, although their efficacy as anti-allergy activity is moderate when compared to pharmaceuticals. These food ingredients could be a way to decrease the severity of some allergic symptoms, such as those related to allergic rhinitis.

**Figure 1:**
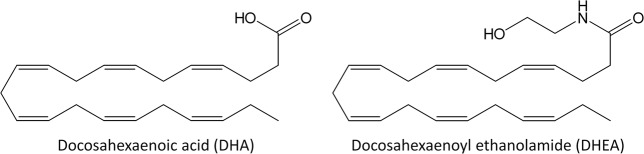
Chemical structures of docosahexaenoic acid (DHA) and docosahexaenoyl ethanolamide (DHEA).

One such food ingredient is fish oil. A number of epidemiological studies have indicated that fish consumption is associated with decreased prevalence of allergies^5,6^. Docosahexaenoic acid or DHA ((4*Z*,7*Z*,10*Z*,13*Z*,16*Z*,19*Z*)-docosa-4,7,10,13,16,19-hexaenoic acid, Fig. 1) is a long-chain polyunsaturated fatty acid mainly found in fish oil, and several studies have suggested that it may be important in the reduction of allergies^6,7^ and have suggested possible mechanisms of action^8,9^; however, the mechanism underlying the anti-allergy effect of DHA still remains to be completely elucidated. Certain DHA metabolites, such as protectin and resolvin produced in the human body, have been reported to promote the resolution of inflammation^10^. In addition, α-linolenic acid and eicosapentaenoic acid, which are also long-chain polyunsaturated fatty acids, have been reported to exert an anti-allergic effect by being converted into 17,18-epoxyeicosatetraenoic acid^11^. We thus explored the anti-allergy activity of DHA metabolites on the stabilization of mast cells, since mast cells, which reside in various tissues, are known to be important key effector cells in IgE-mediated hypersensitivity reactions^3^. After a preliminary screening of several DHA metabolites, we found that DHA does not have a strong suppressive effect on mast cell degranulation but docosahexaenoyl ethanolamide or DHEA ((4*Z*,7*Z*,10*Z*,13*Z*,16*Z*,19*Z*)-*N*-(2-hydroxyethyl)docosa-4,7,10,13,16,19-hexaenamide, Fig. 1), a metabolite produced from DHA in the human body, does. In this study, we evaluated whether DHEA suppresses mast cell degranulation by conducting *in vitro* cell-based assays and *in vivo* mouse models of IgE-mediated hypersensitivity reactions. Consequently, we found that DHEA is a promising agent for alleviating allergic symptoms.

## Results and Discussion

### DHEA suppresses degranulation of RBL-2H3 cells and bone marrow-derived mast cells (BMMCs)

In this study, we focused on the effect of DHEA on mast cells. Mast cells are one of the important targets for alleviating allergic symptoms because they play a critical role in IgE-mediated hypersensitivity reactions^3^. Upon exposure to a multivalent antigen, the IgE-bound high-affinity IgE receptor (FcεRI) on the plasma membrane is crosslinked, which elicits mast cell activation and induces degranulation, leading to the release of various *de novo*‐synthesized and preformed inflammatory mediators including histamine^12^. Histamine released from mast cells results in allergic inflammations such as tissue edema and mucus overproduction^13^. In this study, rat basophilic leukemia RBL-2H3 cells were used for *in vitro* cell-based assays because the cells express FcεRI on their plasma membrane and hence, have been used for investigations on the mechanism of mast cell degranulation^14^.

**Figure 2:**
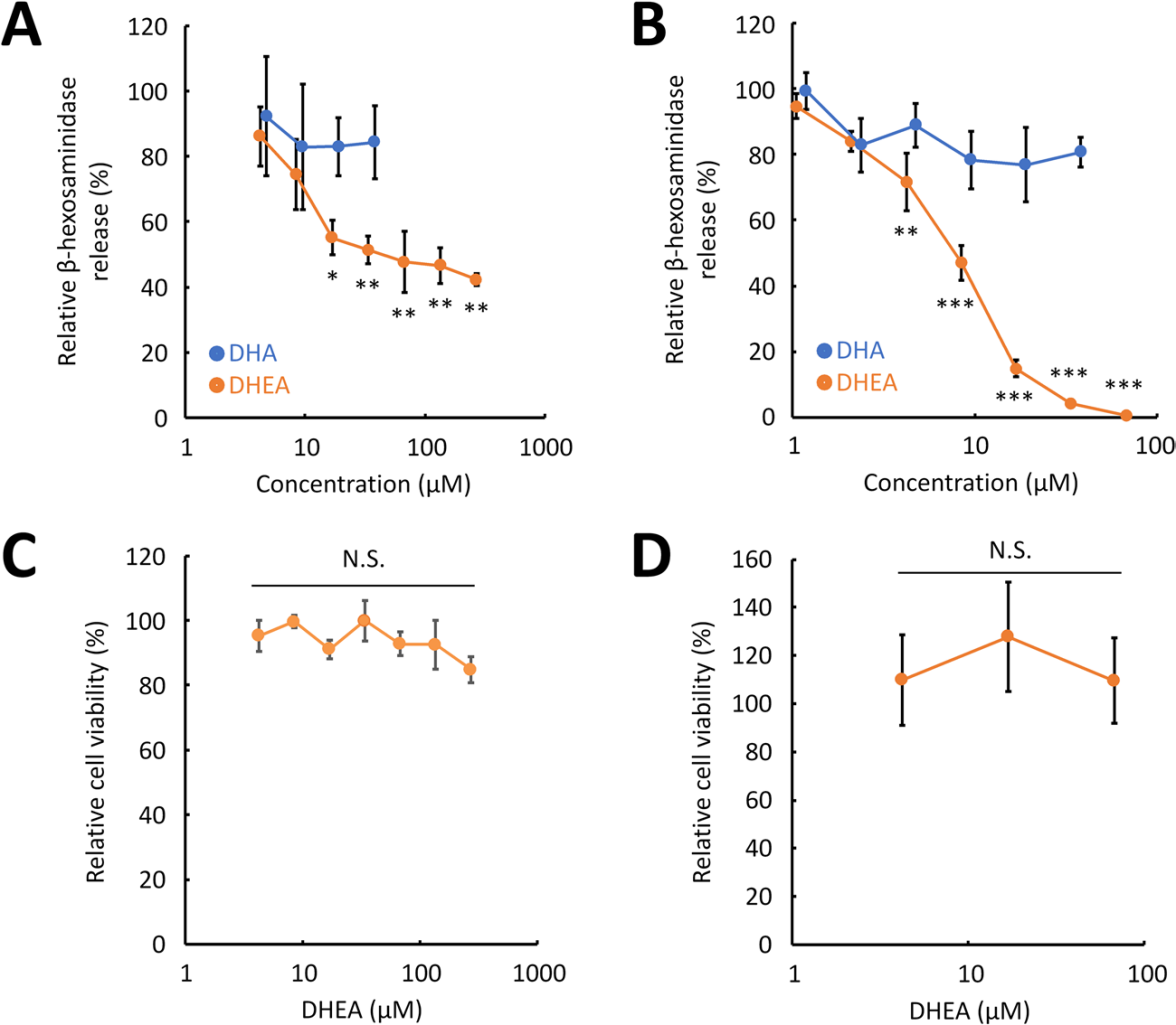
DHEA suppresses degranulation of RBL-2H3 cells and BMMCs without cytotoxicity. The effect of DHEA and DHA on degranulation of RBL-2H3 cells (**A**) and of BMMCs (**B**). Cells sensitized with anti-DNP IgE were treated with various concentrations of DHEA or DHA or with 0.1% ethanol (vehicle). The cells were subsequently stimulated with DNP-HSA, and the enzymatic activity of β-hexosaminidase released from the cells was measured. Relative β-hexosaminidase release was calculated by comparing the enzymatic activity of DHEA-treated cells to that of the cells treated with 0.1% ethanol. The effect of DHEA on the viability of RBL-2H3 cells (**C**) and of BMMCs (**D**). Cells were treated with various concentrations of DHEA or with 0.1% ethanol (vehicle) for 24 h. The WST-8 reagent was then added to the culture medium, and the absorbance was measured. Relative cell viability was calculated by comparing the absorbance obtained from the cells treated with DHEA to that treated with 0.1% ethanol. Data are presented as the mean ± SEM (*n* = 3). Dunnett’s test was used to assess the statistical significance of the difference (^*^P < 0.05, ^**^P < 0.01, ^***^P < 0.001) against control. N.S. indicates not statistically significant.

We at first examined the effect of DHEA and DHA on degranulation of RBL-2H3 cells and BMMCs. Anti-dinitrophenyl (DNP) IgE-sensitized RBL-2H3 cells or BMMCs were treated with various concentrations of DHEA or DHA and subsequently stimulated with DNP-human serum albumin (HSA). The amount of β-hexosaminidase released from cells was then determined by measuring the enzymatic activity which was used as a marker to evaluate the effect of DHEA or DHA on mast cell degranulation. β-Hexosaminidase is released along with histamine upon mast cell degranulation^15^, and the released amount is commonly used as an indicator of degranulation^16^. As shown in Fig. 2A, DHA did not suppress the degranulation of RBL-2H3 cells, or degranulation of BMMCs (Fig. 2B). DHA sodium salt has been previously reported to suppress degranulation of mouse BMMCs^9^, which seems to conflict with our observation. In the previous study, they used a higher concentration of DHA sodium salt (100 µM) for the degranulation assay. Thus, the inconsistency may be caused by different experimental conditions and materials.

Subsequently, the effect of DHEA on degranulation was also examined. DHEA suppressed degranulation of RBL-2H3 cells in a concentration-dependent manner (Fig. 2A), which was statistically significant at 17 µM or higher concentrations. DHEA also suppressed degranulation of BMMCs in a concentration-dependent manner (Fig. 2B), which was statistically significant at 4.2 µM or higher concentrations, thereby showing that BMMCs are more sensitive to DHEA than RBL-2H3 cells. In higher concentrations of DHEA, degranulation was almost completely suppressed in BMMCs (Fig. 2B), whereas degranulation of RBL-2H3 cells was not strongly inhibited (Fig. 2A). This implies that the target protein of DHEA might be less expressed in RBL-2H3 cells than in BMMCs.

The effect of DHEA on the cell viability was then examined. RBL-2H3 cells were treated with various concentrations of DHEA for 24 h, and viable cell number was then estimated using the WST-8 reagent. As shown in Fig. 2C, there was no statistically significant difference in the viable cell number between DHEA-treated and non-treated cells. In addition, BMMCs were treated in the same way as RBL-2H3 cells, and the effect of DHEA on viability of BMMCs was evaluated. As shown in Fig. 2D, no cytotoxic effect of DHEA was found, suggesting that DHEA does not affect the cell viability at any concentration tested in this study. The result showed that the decreased release of β-hexosaminidase from RBL-2H3 cells and BMMCs is not caused by the cytotoxicity, but by the suppressive activity of DHEA on mast cell degranulation.

### DHEA suppresses the increase in the intracellular Ca^2+^ concentration ([Ca^2+^]_i_) in RBL-2H3 cells

**Figure 3:**
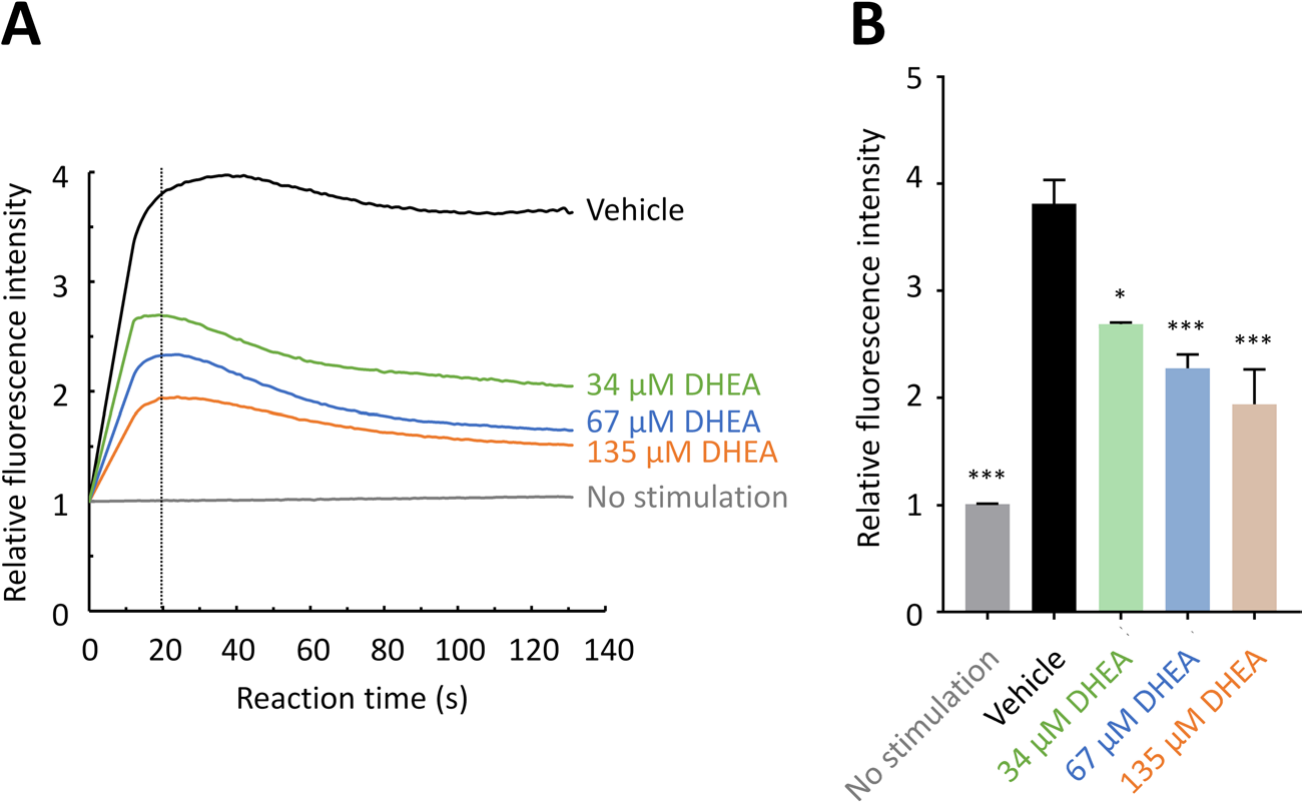
DHEA suppresses the elevation in intracellular Ca^2+^ concentration. RBL-2H3 cells sensitized with anti-DNP IgE were incubated in the medium containing Fluo 4-AM. The cells were then treated with various concentrations of DHEA or with 0.1% ethanol (vehicle), and the fluorescent intensity was immediately monitored upon stimulation with DNP-HSA. Relative fluorescent intensity was calculated by comparing the fluorescent intensity after stimulation to that before stimulation. (**A**) Time course of intracellular Ca^2+^ concentration in RBL-2H3 cells. Data are presented as the mean of triplicate of wells. (**B**) Relative fluorescence intensity at 20 s after antigen stimulation. Data are presented as the mean ± SEM (*n* = 3). Dunnett’s test was used to assess the statistical significance of the difference (^*^P < 0.05, ^***^P < 0.001) against control (vehicle).

FcεRI aggregation initiates signaling cascades leading to the increase in [Ca^2+^]_i_. Elevation in [Ca^2+^]_i_ is a principal signaling for mast cell degranulation^17^. We then examined the effect of DHEA on [Ca^2+^]_i_ in RBL-2H3 cells using a fluorescent calcium indicator Fluo 4-AM. Anti-DNP IgE-sensitized and non-sensitized RBL-2H3 cells were loaded with Fluo 4-AM and then treated with 34, 67, or 135 µM DHEA or with 0.1% ethanol (vehicle) alone. The fluorescence was measured immediately upon the stimulation with DNP-HSA. As shown in Fig. 3A, [Ca^2+^]_i_ increased immediately after stimulation of the vehicle-treated cells with the antigen, while [Ca^2+^]_i_ did not change with no antigen stimulation. The increase in [Ca^2+^]_i_ was suppressed in a concentration-dependent manner when the cells were treated with DHEA. Figure 3B shows the relative fluorescence intensity at 20 s after antigen stimulation. The relative fluorescence intensity was significantly reduced by treating the cells with DHEA, suggesting that DHEA reduces degranulation of RBL-2H3 cells by down-regulating the intracellular signaling cascade upstream of the Ca^2+^ influx.

### DHEA tends to down-regulate the intracellular signaling cascades involved in degranulation

**Figure 4:**
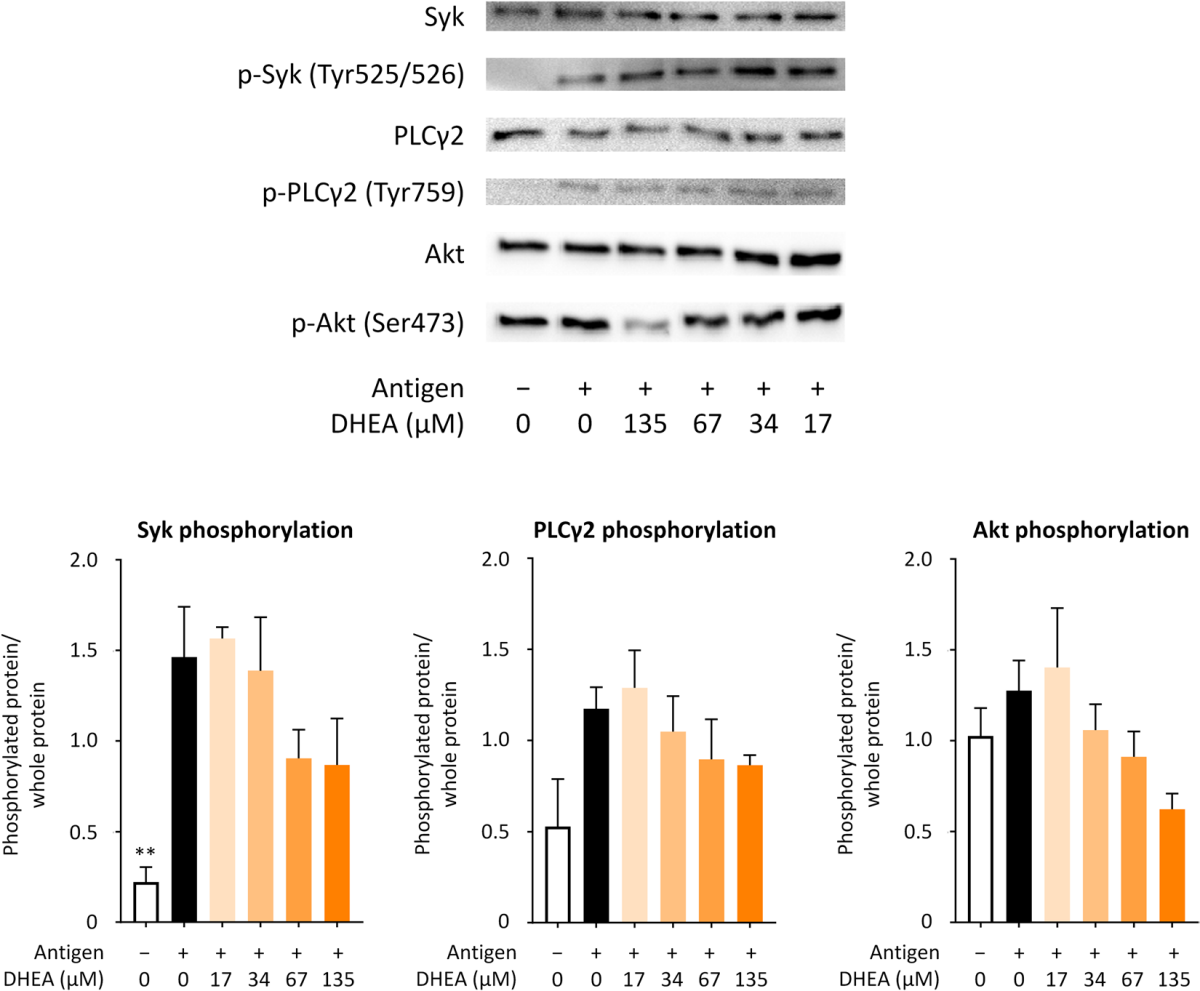
DHEA tends to down-regulate intracellular signaling pathways related to degranulation of RBL-2H3 cells in a dose-dependent manner. RBL-2H3 cells sensitized with anti-DNP IgE were treated with 17, 34, 67, or 135 µM DHEA or with 0.1% ethanol (vehicle) and subsequently stimulated with DNP-HSA for 5 min. Each cell lysate was then prepared to use for immunoblot analysis. p-Syk, p-PLCγ2, and p-Akt indicate phosphorylated Syk (Tyr525/526), phosphorylated PLCγ2 (Tyr759), and phosphorylated Akt (Ser473), respectively. A representative blot from three independent experiments is shown. The ratio of phosphorylation is shown as bar graphs. Data are presented as the mean ± SEM of three independent experiments. Dunnett’s test was used to assess the statistical significance of the difference (^**^P < 0.01) against control (antigen, + ; DHEA, −). The original blots are shown in the supplementary information file.

Because DHEA suppressed the increase in [Ca^2+^]_i_ of RBL-2H3 cells, DHEA was expected to modulate the intracellular signaling cascade involved in mast cell degranulation. We thus examined the effect of DHEA on the phosphorylation state of intracellular signaling proteins by immunoblot analysis. Anti-DNP IgE-sensitized RBL-2H3 cells were treated with 0, 17, 34, 67, or 135 µM DHEA for 30 min and subsequently stimulated with DNP-HSA for 5 min. The cells were then lysed and subjected to immunoblotting. The results showed that DHEA indicated a tendency to suppress the phosphorylation of Syk, phospholipase C-γ2 (PLCγ2), and Akt in a dose-dependent manner (Fig. 4). IgE-mediated FcεRI aggregation caused by cross-linking antibodies to antigens provokes intracellular signaling cascades leading to mast cell degranulation. Syk tyrosine kinase plays an essential role in mast cell degranulation and is activated immediately upon FcεRI stimulation, followed by the activation of PLCγ and Akt pathways^18,19^. PLCγ also plays a crucial role in the production of inositol 1,4,5-trisphosphate and in regulating intracellular Ca^2+^ mobilization, which leads to mast cell degranulation^20^. Inhibited phosphorylation of Syk and PLCγ2 has been shown to abolish FcεRI-mediated mast cell degranulation^21,22^. Akt is located downstream of Syk and is involved in the mast cell degranulation^23^. Overall, these results indicate that the suppressive effect of DHEA on mast cell degranulation might arise from the inhibition of Syk, which subsequently results in the down-regulation of Syk-PLCγ and Syk-Akt pathways, leading to reduced influx of Ca^2+^ into the cells and prevention of IgE-mediated degranulation of RBL-2H3 cells; however, statistical significance of the difference between DHEA-treated and non-treated cells was not observed. The reason might be that DHEA does not exert the degranulation-suppressive effect enough at the tested concentrations because the effect of DHEA is less potent in RBL-2H3 cells than in BMMCs in higher concentrations (Fig. 2A,B). In this study the suppressive activity/mechanism of DHEA is not addressing all signaling processes necessary for degranulation and thus under investigation.

### DHEA attenuates IgE-mediated passive cutaneous anaphylaxis (PCA) reaction *in vivo*

**Figure 5:**
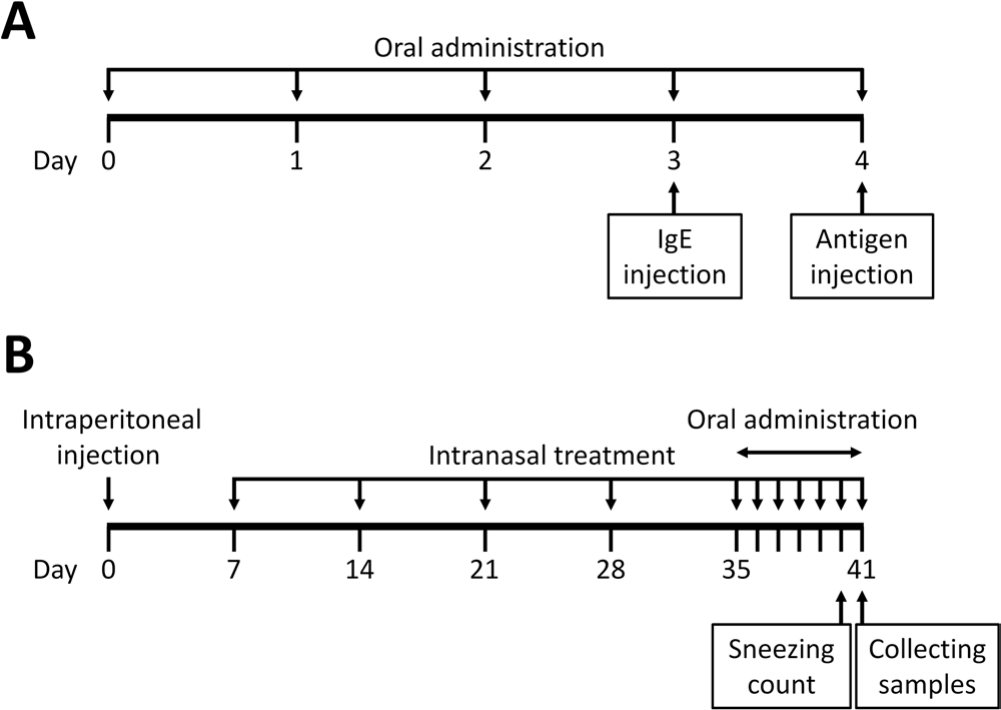
Schematic depiction of the experimental schedule to induce allergic reactions. (**A**) PCA reaction in a mouse model. (**B**) A mouse model of Japanese cedar pollinosis.

**Figure 6:**
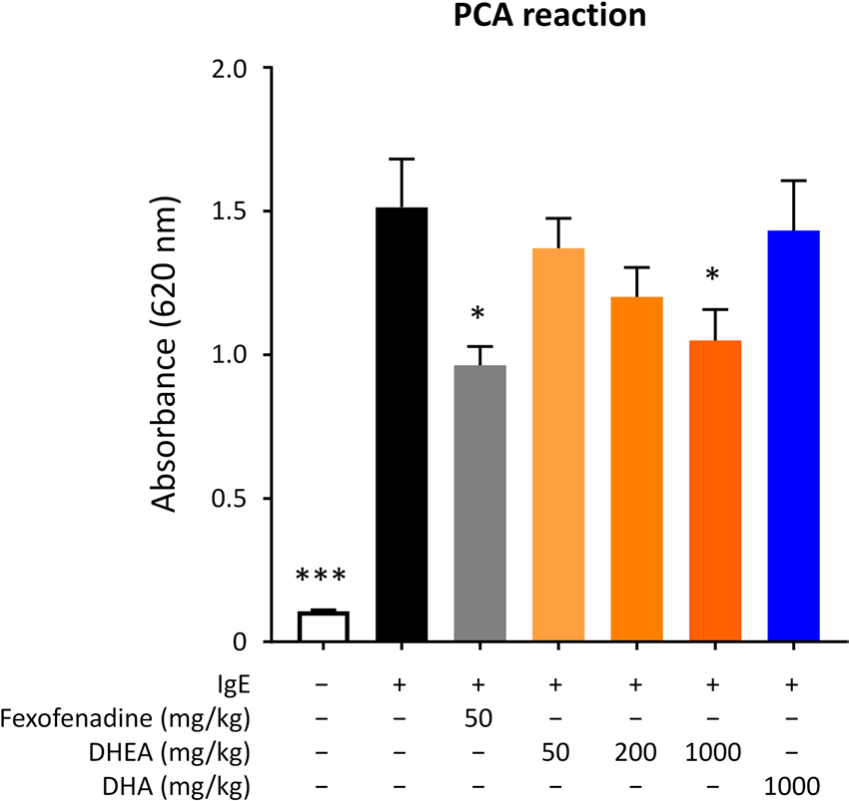
DHEA suppresses PCA reaction in a mouse model. Mice were orally administered DHEA, DHA, fexofenadine hydrochloride (positive control), or water (intact and control groups) for 5 consecutive days. One day before the final oral administration, an ear of mice was intradermally injected with anti-DNP IgE. One hour after the final oral administration, all mice were intravenously injected with DNP-HSA and Evans blue dye. After 30 min, the ear was excised, and the absorbance of the dye was measured. Data are presented as the mean ± SEM (*n* = 7–8). Dunnett’s test was used to assess the statistical significance of the difference (^*^P < 0.05, ^***^P < 0.001) against control.

To determine whether DHEA could also suppress degranulation *in vivo*, we examined the *in vivo* activity of DHEA using the PCA reaction in mice. PCA is a localized cutaneous allergic response resulting from vascular hyperpermeability and plasma extravasation following the allergen exposure^24^ and is used as an animal model of IgE-mediated allergic response to evaluate the effect of bioactive molecules^25^. Because a large amount of DHEA was needed for animal experiments, we synthesized DHEA as described in the “Materials and Methods” section. Mice were administered DHEA at the dose of 50 mg/kg, 200 mg/kg, or 1,000 mg/kg, DHA at 1,000 mg/kg, fexofenadine hydrochloride at 50 mg/kg, or water at 1,000 mg/kg for 5 consecutive days from day 0 to day 4. With the exception of the intact group, mice were intradermally injected with anti-DNP IgE in an ear on day 3, and then all mice were intravenously injected with DNP-HSA and Evans blue dye on day 4 as shown in Fig. 5A. The absorbance of the dye in the tissue after extravasation was then measured. As shown in Fig. 6, the absorbance of the dye extracted from IgE-sensitized mice was much higher than that from non-sensitized mice, indicating that the PCA reaction occurred successfully. Fexofenadine hydrochloride, a selective histamine H_1_ receptor antagonist, was used as a positive control and significantly abolished PCA reaction. In addition, DHEA appeared to suppress the PCA reaction in a dose-dependent manner; only the highest dose used (1,000 mg/kg) yielded a significant result. DHA showed almost no effect on PCA test, corroborating that DHA itself did not have a suppressive effect on mast cell degranulation (Fig. 2A,B). The result suggested that, in mice, the 5-day intake of DHEA is effective in suppression of mast cell degranulation *in vivo*.

### DHEA and DHA alter biochemical parameters in a model of pollinosis

**Figure 7:**
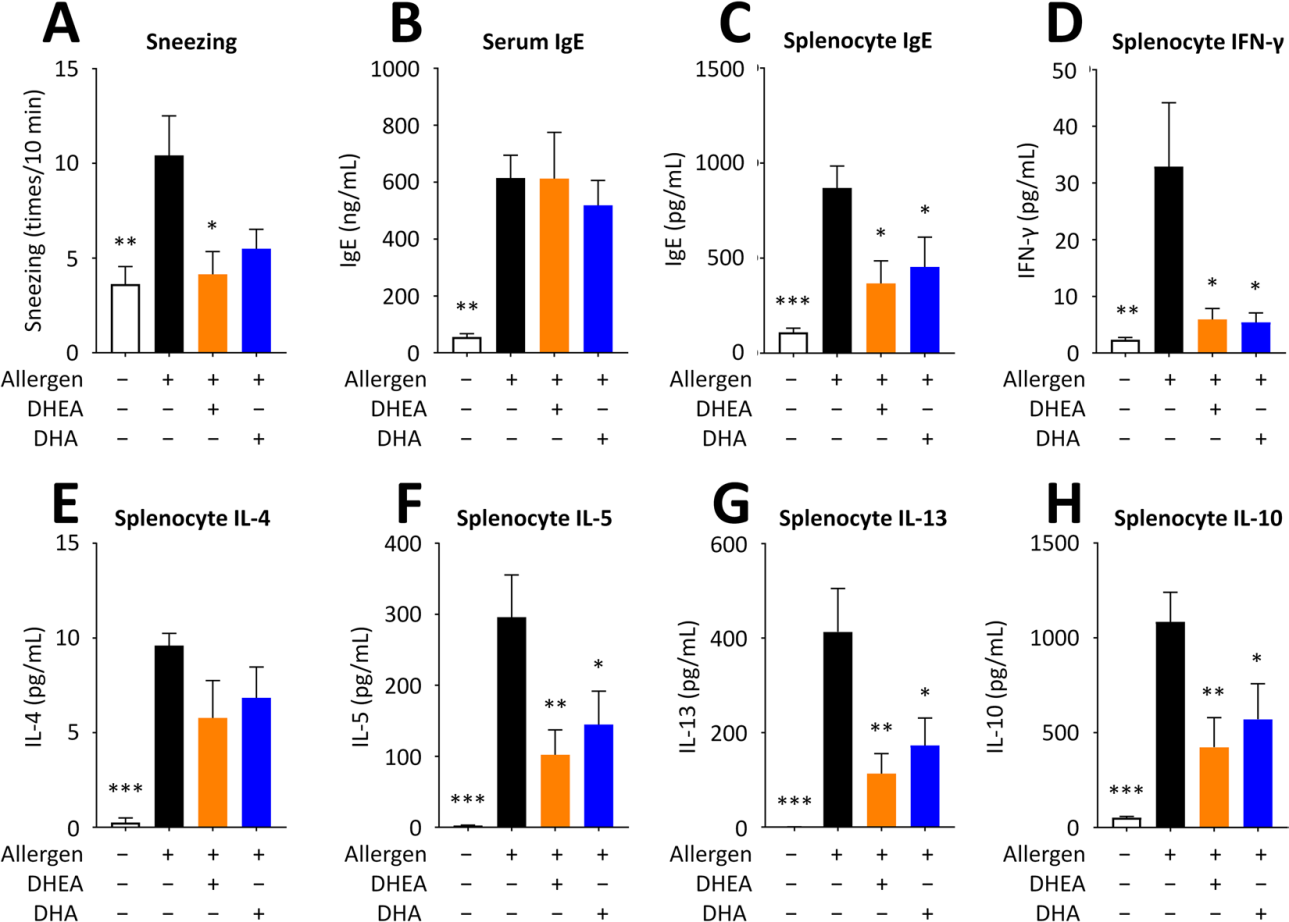
DHEA mitigates an allergic symptom of Japanese cedar pollinosis and alters the production of IgE and cytokines secreted by splenocytes collected from pollinosis mice. Mice were intraperitoneally injected on day 0 and intranasally treated on day 7, 14, 21, 28, and 35 to 39 with Cry j1 and Cry j2 (Japanese cedar pollen allergen). Mice in DHEA and DHA groups were dosed with DHEA and DHA, respectively, from day 35 to day 41, while control and intact groups were with water. On day 40, all mice were intranasally treated with Cry j1 and Cry j2, and the sneezing frequency was counted for 10 min. On day 41, the blood and spleen were collected. (**A**) Sneezing frequency of pollinosis mice after intranasal challenge with the allergen. (**B**) Serum IgE levels of pollinosis mice. Production of IgE (**C**), IFN-γ (**D**), IL-4 (**E**), IL-5 (**F**), IL-13 (**G**), and IL-10 (**H**) by splenocytes collected from pollinosis mice. Data are presented as the mean ± SEM (*n* = 8). Dunnett’s test was used to assess the statistical significance of the difference (^*^P < 0.05, ^**^P < 0.01, ^***^P < 0.001) against control.

Japanese cedar (*Cryptomeria japonica*) pollen is one of the most common sources of allergens that elicit seasonal allergic rhinitis in Japan. We thus evaluated the effect of DHEA on allergic symptoms caused by Japanese cedar pollinosis using a mouse model. All mice, except intact group, were sensitized with Cry j1 and Cry j2 proteins, which are the major allergen contained in Japanese cedar pollens, as scheduled in Fig. 5B. Mice were orally administered DHEA at the dose of 1,000 mg/kg (DHEA group), DHA at 1,000 mg/kg (DHA group), or water (intact and control groups) at 1,000 mg/kg for seven consecutive days, and then an allergic symptom and serum IgE level were evaluated. The results showed that sneezing frequency of the control mice significantly increased compared with that of the intact mice (Fig. 7A). In addition, serum IgE level of the control mice was significantly higher than that of the intact mice (Fig. 7B). These data indicate that a mouse model of pollinosis was successfully established by sensitizing mice with Cry j1 and Cry j2. Intake of DHEA significantly reduced the sneezing frequency of mice compared with that of control mice (Fig. 7A), suggesting that DHEA blocks the histamine release from mast cells located in the nasal mucosa by suppressing degranulation. Interestingly, oral administration of DHA tended to reduce the sneezing frequency (*P* = 0.0677 against control) in spite of almost no effect in the PCA test, suggesting that DHA metabolites, not DHA itself, might exert a suppressive effect on mast cell degranulation or that DHA affects certain immune cells other than mast cells to alleviate allergic symptoms. Further investigation is now being conducted to determine the specific causes of this observation. The serum IgE level of the DHEA-administered and DHA-administered mice remained unchanged compared with that of the control mice (Fig. 7B). This result indicates that the mitigation effect of DHEA or DHA on the allergic symptom is not attributed to the decreased IgE production.

Because DHA appears to ameliorate allergic symptoms by regulating immune cells other than mast cells, we further explored the effect of DHEA and DHA by examining the responses of splenocytes collected from the Cry j1- and Cry j2-sensitized mice after evaluating their effect on the sneezing frequency. The splenocytes were cultured in the presence of allergen (Cry j1 and Cry j2) for 96 h after isolation, and the concentration of cytokines and IgE in the culture medium was measured. The results showed that the production of IgE (Fig. 7C), interferon-γ (IFN-γ; Fig. 7D), interleukin-5 (IL-5; Fig. 7F), IL-10 (Fig. 7G), and IL-13 (Fig. 7H) by splenocytes was significantly suppressed by oral administration of DHEA or DHA. In addition, the secretion of IL-4 tended to reduce, however not significantly, by intake of DHEA (*P* = 0.1066 against control; Fig. 7E) or of DHA (*P* = 0.3055). IL-4, IL-5, and IL-13 are classified as Th2 cytokines^26^. IL-4 and IL-13, in particular, are produced by T cells, mast cells, and basophils and known to induce class switching to IgE and gene expression of FcεRI, IgE, inflammatory cytokines, and chemokines^27,28^. Thus, DHEA and DHA seemed to affect T cells and mast cells to decrease production of IL-4 and IL-13, which leads to alleviation of allergic reactions. Because DHEA and DHA reduced the production of IL-5 and IL-13 greater than that of IL-4, it may affect the group 2 innate lymphoid cells, which secret IL-5 and IL-13 rather than IL-4^29^. Some bioactives, such as gallic acid^30^ and skullcapflavone II^31^, attenuate allergic responses by reducing the expression of Th2 cytokines, such as IL-4, and by increasing Th1 cytokines, such as IFN-γ^32,33^; however, DHEA or DHA did not increase the production of Th1 cytokine IFN-γ (Fig. 6D). Thus, the mechanism of anti-allergic activity of DHEA and DHA is not to improve the Th1/Th2 balance. DHEA and DHA also significantly reduced the production of IL-10 (Fig. 6H), which is known to be secreted by regulatory T cells to suppress allergic inflammation^34^; however, some studies have demonstrated that IL-10 might have a facilitative effect on the development of allergy^35,36^. Takagi *et al*. (2005) demonstrated that the immunotherapy using allergen-specific T cell epitope peptides for inducing oral tolerance to pollinosis results in reduced production of IL-4, IL-5, IL-13, IL-10, and IFN-γ^37^. In addition, they showed reduced sneezing frequency, but unaffected serum IgE level in a mouse model of pollinosis. Their results support what we have observed in this study. Hence, the mechanism of anti-allergy activity of DHEA and DHA might be similar to inducing oral tolerance to an allergen. In Fig. 7, DHA was consistently less effective than DHEA in spite of being given at the same dose. The reason might be that DHA has less effect on T cells or other immune cells than DHEA or that the serum level of DHA is more readily reduced than that of DHEA after intake by being incorporated to membrane lipids in our body.

In conclusion, our study has demonstrated that DHEA is a promising agent for mitigating IgE-mediated allergic reactions. *In vitro* experiments revealed that DHEA suppresses degranulation of RBL-2H3 cells and BMMCs without cytotoxicity by affecting intracellular signaling pathways related to mast cell degranulation. The PCA experiment indicated that the intake of DHEA could suppress mast cell degranulation *in vivo*. The experiment using a mouse model of Japanese cedar pollinosis suggested that DHEA could mitigate allergic reactions by affecting not only mast cells but also other immune cells and that oral administration of DHA is effective in alleviating allergic reactions of pollinosis, although DHA is less effective than DHEA. It is still uncertain whether the anti-allergy effect of DHA comes from DHA itself or from DHEA that is converted from DHA after intake. Further investigation on the anti-allergy effect of DHA is now being conducted. Taken together, this study indicates that DHEA is a promising anti-allergic agent by inhibiting mast cell degranulation and by modulating other immune cells to mitigate allergic reactions.

## Materials and Methods

### Reagents

DHEA and DHA dissolved in ethanol were purchased from Cayman Chemical (Ann Arbor, Michigan, USA) and used for cell-based assays shown below. Dulbecco’s modified Eagle’s medium (DMEM), RPMI 1640 medium, fetal bovine serum (FBS), penicillin, streptomycin, bovine serum albumin (BSA), mouse anti-DNP monoclonal IgE, DNP-HSA conjugate, Triton X-100, and Evans blue dye were purchased from Sigma-Aldrich (St. Louis, MO, USA). Recombinant murine IL-3 and murine stem cell factor (SCF) were purchased from PeproTech (Rocky Hill, NJ, USA). Phycoerythrin (PE)-labelled anti-mouse CD117 and fluorescein isothiocyanate (FITC)-labelled anti-mouse FcεRIα were purchased from BioLegend (San Diego, CA, USA). Horseradish peroxidase (HRP)-labelled anti-rabbit IgG antibody and rabbit antibodies against Syk (#13198), phosphorylated Syk at Tyr525/526 (#2710), Akt (#4691), phosphorylated Akt at Ser473 (#4060), PLCγ2 (#3872), and phosphorylated PLCγ2 at Tyr759 (#3874) were purchased from Cell Signaling Technology (Danvers, MA, USA). Aluminium hydroxide gel was purchased from Fujifilm Wako Pure Chemical (Osaka, Japan). Fexofenadine hydrochloride was purchased from Tokyo Chemical Industry (Tokyo, Japan). Purified Japanese cedar pollen protein (Cry j1 and Cry j2) was purchased from Hayashibara (Okayama, Japan). An enzyme-linked immunosorbent assay (ELISA) kit to measure the IgE concentration was obtained from BioLegend (San Diego, CA, USA). ELISA kits to measure the concentration of IL-4, IL-5, IL-10, IL-13, and IFN-γ were obtained from Invitrogen (Carlsbad, CA, USA). All other chemicals were purchased from Fujifilm Wako Pure Chemical or Nacalai Tesque (Kyoto, Japan) unless otherwise noted.

### Cells and cell culture

RBL-2H3 cells were obtained from the American Type Culture Collection (Rockvile, MD, USA) and cultured in DMEM supplemented with 100 U/mL of penicillin, 100 µg/mL of streptomycin, and 10% heat-inactivated FBS at 37 °C under humidified 5% CO_2_ in air.

To generate BMMCs, bone marrow cells were harvested from femurs of a BALB/c mouse at the age of 8 weeks by repeated flushing with phosphate-buffered saline (PBS, pH 7.4) and cultured in RPMI 1640 medium supplemented with 10% FBS, 2 mM L-glutamine, 1 mM pyruvate, 100 U/mL of penicillin, 100 μg/mL of streptomycin, and 4 ng/mL of murine IL-3. Nonadherent cells were transferred to fresh medium 3 d after cell harvest and differentiated into BMMCs over 4 weeks under the influence of IL-3 at the density of 1.0 × 10^6^ cells/mL. SCF was applied at 40 ng/ml for the last 2 weeks. The differentiation status of BMMCs was analyzed using flow cytometry (BD FACSVerse, BD Biosciences, Franklin Lakes, NJ, USA) by staining with PE-labelled anti-mouse CD117 and with FITC-labelled anti-mouse FcεRIα. The purity of BMMCs was confirmed to be higher than 90%.

### β-Hexosaminidase release assay

The assay was performed as previously reported^38^ with some modifications. RBL-2H3 cells or BMMCs were seeded at 4.0 × 10^4^ cells/well in a 96-well culture plate (Corning, Corning, NY, USA) and sensitized with anti-DNP IgE (50 ng/mL) at 37 °C overnight. After washing the cells with modified Tyrode’s (MT) buffer (20 mM HEPES, 135 mM NaCl, 5 mM KCl, 1.8 mM CaCl_2_, 1 mM MgCl_2_, 5.6 mM glucose, and 0.05% BSA, pH 7.4) twice, the cells were treated with 120 μL of MT buffer containing various concentrations of DHEA or 0.1% (v/v) ethanol (vehicle) for 30 min at 37 °C. The cells were then stimulated with 10 μL of DNP-HSA (625 ng/mL) for 30 min at 37 °C. After stimulation, the supernatant was collected from each well, and the cells were sonicated in 130 μL of MT buffer containing 0.1% Triton X-100 for 5 s on ice. Both of the supernatant and cell lysate were transferred to a new 96-well microplate at 50 μL/well and incubated for 5 min at 37 °C. One hundred μL of 3.3 mM 4-nitrophenyl 2-acetamido-2-deoxy-β-D-glucopyranoside dissolved in 0.1 M citrate buffer (pH 4.5) were then added to each well and incubated for 25 min at 37 °C. The enzyme reaction was terminated by adding 100 µL of 2 M glycine buffer (pH 10.4), and the absorbance was measured at 405 nm using a microplate reader (SH-8000Lab; Corona Electric, Hitachinaka, Japan). A β-hexosaminidase release rate (%) was calculated as follows:

$${\rm{100}}\times [\frac{{(A}_{{\rm{supernatant}}}-{{\rm{A}}}_{{\rm{blank}}{\rm{of}}{\rm{supernatant}}})}{\{({{\rm{A}}}_{{\rm{supernatant}}}-{{\rm{A}}}_{{\rm{blank}}{\rm{of}}{\rm{supernatant}}})+({{\rm{A}}}_{{\rm{cell}}{\rm{lysate}}}-{{\rm{A}}}_{{\rm{blank}}{\rm{of}}{\rm{cell}}{\rm{lysate}}})\}}]$$

in which “A” is the absorbance of each well.

### Cell viability assay

Toxicity of DHEA to RBL-2H3 cells or to BMMCs was evaluated using Cell Count Reagent SF (Nacalai Tesque) containing 2-(2-methoxy-4-nitrophenyl)-3-(4-nitrophenyl)-5-(2,4-disulfophenyl)-2H-tetrazolium or WST-8 according to the manufacturer’s instructions. After cells were seeded into each well of a 96-well culture plate at 1.0 × 10^4^ cells/well and cultured overnight, the cells were treated with 100 μL of DMEM containing various concentrations of DHEA or 0.1% (v/v) ethanol (vehicle) for 24 h at 37 °C. The culture medium was then aspirated, and 100 μL of DMEM containing 10% (v/v) Cell Count Reagent SF were added to each well of the 96-well microplate. The WST-8 formazan dye intensity was then monitored at the wavelength of 450 nm in a microplate reader (SH-8000Lab).

### Measurement of intracellular calcium level

Intracellular calcium levels were measured using a Calcium kit Fluo 4 (Dojindo Laboratories, Mashiki, Japan) according to the manufacturer’s instructions. RBL-2H3 cells were seeded at 4.0 × 10^4^ cells/well in a 96-well black culture plate (Sumitomo Bakelite, Tokyo, Japan) and sensitized with anti-DNP IgE (50 ng/mL) at 37 °C overnight. After washing with PBS, the cells were incubated in 100 μL of Fluo 4-AM for 1 h at 37 °C. After washing with PBS, the cells were treated with 100 μL of the recording buffer containing 34, 67, or 135 µM DHEA or 0.1% (v/v) ethanol (vehicle) and incubated at 37 °C for 30 min. The cells were then stimulated with DNP-HSA (50 ng/mL), and the fluorescent intensity was immediately monitored with an excitation wavelength of 485 nm and an emission wavelength of 535 nm using a microplate reader (Infinite 200 PRO, Tecan, Männedorf, Switzerland).

### Immunoblot analysis

RBL-2H3 cells were seeded into 35 mm dishes (Corning) at 4.0 × 10^5^ cells/well and sensitized with anti-DNP IgE (50 ng/mL) in DMEM containing 10% FBS at 37 °C overnight. After washing, the cells were treated with 17, 34, 67, or 135 µM DHEA or with 0.1% (v/v) ethanol (vehicle) for 30 min at 37 °C. The cells were then stimulated with DNP-HSA (50 ng/mL) and further incubated for 5 min. After removing the added reagents, cells were immediately lysed and immunoblotting was performed as previously reported^39^. Blots were developed by ImmunoStar LD (Fujifilm Wako Pure Chemical). Bands were visualized using a ChemiDoc XRS Plus apparatus (Bio-Rad Laboratories, Hercules, CA, USA), and the chemiluminescent intensity was quantified using the Quantity One software (Bio-Rad Laboratories).

### Animals

Female BALB/c mice were purchased from Clea Japan (Tokyo, Japan) and housed in a temperature-controlled (24 ± 2 °C) environment under 12 h light/dark cycle. Animals received standard chow (Rodent LabDiet EQ. 5L37; Nutrition International, Brentwood, MO, USA) and water *ad libitum*.

### Synthesis of DHEA

For synthesis of DHEA, DHA was purchased from Nu-Chek Prep (Elysian, MN, USA). ^1^H-NMR and ^13^C-NMR spectra were recorded on a JEOL JNM-EX400 spectrometer (JEOL, Japan) using tetramethylsilane (TMS) as an internal standard. High resolution mass spectra were measured by liquid chromatography/electrospray ionization quadrupole time-of-flight mass spectrometry (LC/ESI-Q-Tof-MS) (Acquity UPLC/Xevo QTof MS system; Waters, Milford, MA, USA) in the positive ion mode. Thin-layer chromatography (TLC) was performed on silica gel, and spots were visualized with basic KMnO_4_.

To a solution of DHA (0.30 g, 0.91 mmol), 4-dimethylaminopyridine (0.13 g, 1.09 mmol), and 2-aminoethanol (0.082 g, 1.09 mmol) in dry dichloromethane (7 mL) was added 1-[3-(dimethylamino)propyl]-3-ethylcarbodiimide hydrochloride (0.21 g, 1.09 mmol) at room temperature. The reaction mixture was stirred overnight at room temperature under nitrogen gas. The product was extracted with ethyl acetate (50 mL × 2). The combined organic solutions were washed with an aqueous solution of 1 N hydrochloric acid (15 mL) and brine (70 mL × 2), dried over magnesium sulfate, and evaporated to dryness. The residue was purified by using silica gel column chromatography eluting ethyl acetate to afford DHEA in 92% yield. ^1^H-NMR δ (CDCl_3_): 0.97 (3 H, t, *J* = 7.2 Hz), 2.07 (2 H, quint, *J* = 7.2 Hz), 2.27 (2 H, t, *J* = 7.2 Hz), 2.42 (2 H, q, *J* = 7.2 Hz), 2.65 (1 H, s), 2.80–2.88 (10 H, m), 3.43 (2 H, q, *J* = 7.2 Hz), 3.72 (2 H, t, *J* = 7.2 Hz), 5.25–5.44 (12 H, m), 5.95 (1 H, s). ^13^C NMR δ (CDCl_3_): 14.27, 20.53, 23.35, 25.51, 25.60, 36.30, 42.47, 62.56, 126.97, 127.83, 127.97, 128.03, 128.25, 128.30, 128.55, 129.46, 132.03, 173.64. HRMS (ESI) m/z calcd for C_24_H_37_NO_2_ [M + H]^+^ 372.2903, found [M + H]^+^ 372.2919. The purity of DHEA was confirmed to be higher than 95% by HPLC analysis.

### IgE-mediated PCA in mice

An IgE-dependent PCA experiment was performed as previously reported^40^ with some modifications. After acclimating to their housing environment for 1 week, 8-week-old female BALB/c mice were randomly divided into 6 groups (7 or 8 mice per group) and orally administered 50, 200, or 1,000 mg/kg of DHEA, 1,000 mg/kg of DHA, 50 mg/kg of fexofenadine hydrochloride^41^ suspended in 0.5% methyl cellulose (Fujifilm Wako Pure Chemical), or 1,000 mg/kg of water (intact and control groups) once a day for 5 consecutive days (day 0 to day 4) as shown in Fig. 5A. On day 3, all mice were intradermally injected with 10 µL of PBS containing 0.1 μg of anti-DNP IgE into an ear. On day 4, 200 μL of PBS containing 0.2 mg of DNP-HSA and 0.5% (w/v) Evans blue dye were intravenously injected 1 h after oral administration. The mice were euthanized 30 min after intravenous injection, and their ears were collected. The dye in ear tissue after extravasation was then extracted from each ear with 400 μL of formamide at 70 °C overnight, and the absorbance was measured at 620 nm using an Ultrospec 3000 spectrophotometer (Amersham Pharmacia Biotech, Uppsala, Sweden).

### A mouse model of Japanese cedar pollinosis

A mouse model of Japanese cedar pollinosis was developed by treating mice with the purified Japanese cedar pollen protein (Cry j1 and Cry j2) as scheduled in Fig. 5B. Following adaptation period for 1 week, 6-week-old female BALB/c mice were randomly divided into 4 groups as follows: intact group, control group, DHEA-administered group, and DHA-administered group (8 mice/group). All mice, except intact group, were intraperitoneally injected with 5 µg of Cry j1 and Cry j2 absorbed on 2 mg of aluminium hydroxide gel on day 0 and intranasally treated with 2 μg of Cry j1 and Cry j2 on day 7, 14, 21, and 28 and with 0.4 μg of Cry j1 and Cry j2 on day 35 to day 39. DHEA-administered and DHA-administered groups were dosed with 1,000 mg/kg of DHEA and of DHA, respectively, for 7 consecutive days from day 35 to day 41, while control and intact groups were orally administered 20 µL of water. On day 40, all mice, including intact group, were challenged by intranasally treating with 0.4 μg of Cry j1 and Cry j2 1 h after oral administration, and the sneezing frequency was subsequently counted for 10 min. On day 41, the blood and spleen were collected 2 h after the final challenge to all mice. Splenocytes were prepared by gently passing the spleen through a cell strainer (BD Falcon, Franklin Lakes, NJ, USA), hemolyzing with a lysis buffer (155 mM NH_4_Cl, 15 mM NaHCO_3_, 1 mM ethylenediaminetetraacetic acid, pH 7.3), and washing with PBS. The cells were then suspended in RPMI 1640 medium supplemented with 100 U/mL of penicillin, 100 µg/mL of streptomycin, 10% FBS, and 2 µg/mL of Cry j1 and Cry j2 and seeded into 6-well culture plates at 2.0 × 10^7^ cells/well. After cultivation for 96 h at 37 °C, the concentrations of IgE and cytokines in the culture medium were determined using ELISA kits.

### Statistical analysis

Statistical analyses were performed using GraphPad Prism version 7.02 (GraphPad Software, La Jolla, CA, USA). Statistical significance was determined via one-way analysis of variance (ANOVA) with Dunnett’s multiple comparison test as indicated. Values with ^*^P < 0.05, ^**^P < 0.01, or ^***^P < 0.001 against control were considered statistically significant.

### Ethical approval

Protocols of all animal experiments were approved by the Animal Experiment Committee of Ehime University. All animal experiments were carried out in accordance with the Guidelines of Animal Experiments of Ehime University.

## Supplementary information

Supplementary figure.
