# Supplementary material for: Docosahexaenoyl ethanolamide mitigates IgE-mediated allergic reactions by inhibiting mast cell degranulation and regulating allergy-related immune cells
Source: Sci Rep. 2019 Nov 7;9:16213. doi: 10.1038/s41598-019-52317-z (PMC6838076; doi:10.1038/s41598-019-52317-z)
Supplement: Supplementary file 1 — Supplementary figure. [file 41598_2019_52317_MOESM1_ESM.pdf]

Supplementary Information

Docosahexaenoyl ethanolamide mitigates IgE-mediated allergic reactions by inhibiting mast cell degranulation and regulating allergy-related immune cells

Kosuke Nishi[1,2,3*], Yoshiki Kanayama[1], In-Hae Kim[1], Akihiro Nakata[1,4], Hisashi Nishiwaki[1] & Takuya Sugahara[1,2]

[1]Department of Bioscience, Graduate School of Agriculture, Ehime University, Matsuyama, Ehime 790-8566, Japan. [2]Food and Health Sciences Research Center, Ehime University, Matsuyama, Ehime 790-8566, Japan. [3]Research Unit for Skeletal Health and Diseases, Ehime University, Toon, Ehime 791-0295, Japan. [4]Department of Pathophysiology, Graduate School of Medicine, Ehime University, Toon, Ehime, Japan.

*Correspondence and requests for materials should be addressed to K.N. (email: nishi.kosuke.mx@ehime-u.ac.jp)

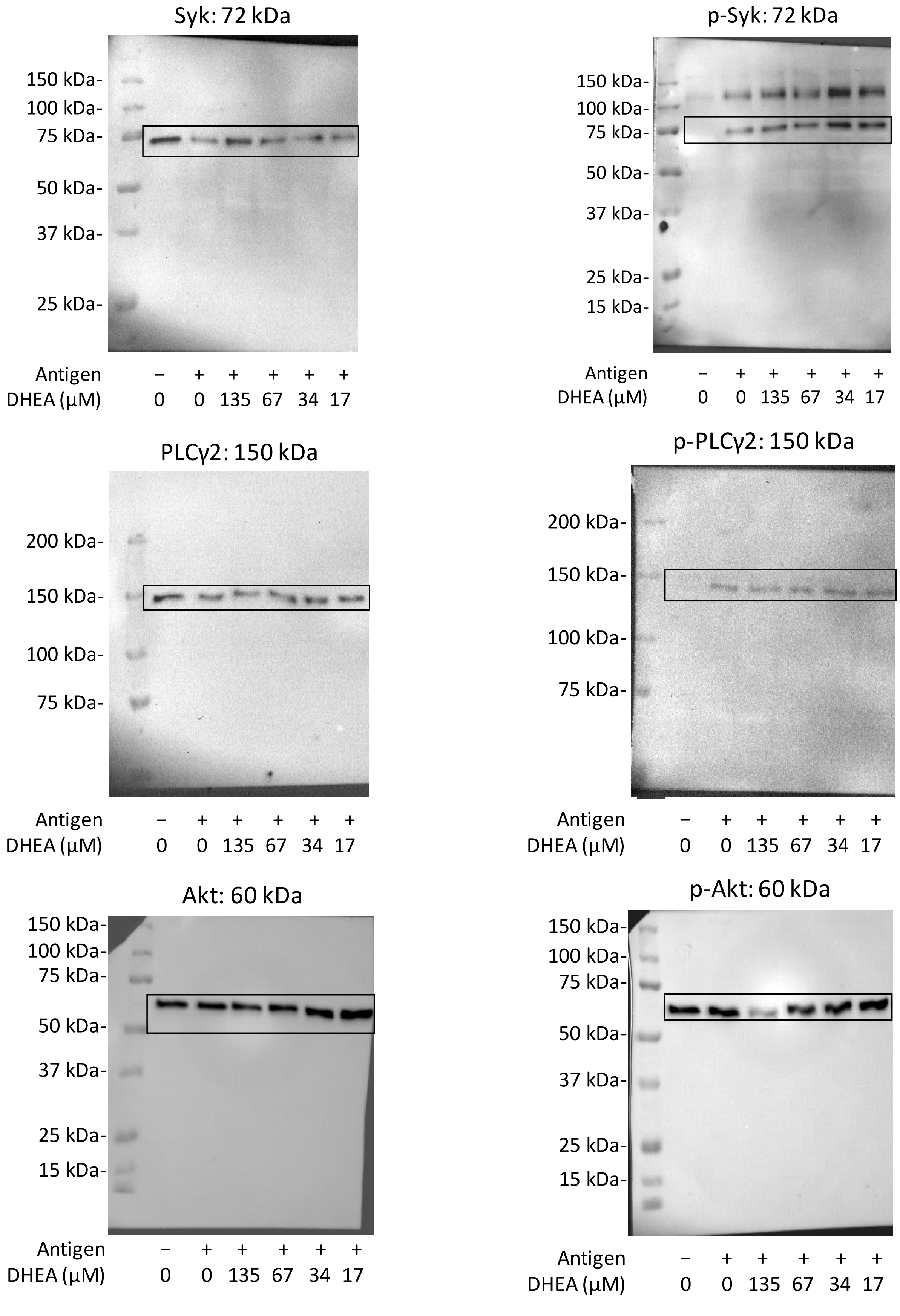

Supplementary figure. Uncropped immunoblot used in Fig. 4.
